# Metabolic regulation of neurodifferentiation in the adult brain

**DOI:** 10.1007/s00018-019-03430-9

**Published:** 2020-01-07

**Authors:** Camilla Maffezzini, Javier Calvo-Garrido, Anna Wredenberg, Christoph Freyer

**Affiliations:** 1 Max Planck Institute Biology of Ageing-Karolinska Institutet Laboratory, Karolinska Institutet, Stockholm, Sweden; 2 Department of Medical Biochemistry and Biophysics, Karolinska Institutet, Stockholm, Sweden; 3 Department of Molecular Medicine and Surgery, Karolinska Institutet, Stockholm, Sweden; 4 Centre for Inherited Metabolic Diseases, Karolinska University Hospital, Stockholm, Sweden; 5 Present Address: Stem Cell and Neurogenesis Unit, Division of Neuroscience, San Raffaele Scientific Institute, 20132 Milan, Italy

**Keywords:** Metabolic switch, Neural stem cells, Neural progenitor cells, Adult neurogenesis, Metabolism, Mitochondria

## Abstract

Understanding the mechanisms behind neurodifferentiation in adults will be an important milestone in our quest to identify treatment strategies for cognitive disorders observed during our natural ageing or disease. It is now clear that the maturation of neural stem cells to neurones, fully integrated into neuronal circuits requires a complete remodelling of cellular metabolism, including switching the cellular energy source. Mitochondria are central for this transition and are increasingly seen as the regulatory hub in defining neural stem cell fate and neurodevelopment. This review explores our current knowledge of metabolism during adult neurodifferentiation.

## Introduction

The potential for pluripotent cells to undergo both replication and differentiation into highly specialised tissues is the central defining feature of multicellular organisms. It also serves as a mean to maintain and repair themselves, and loss of this cellular renewal capacity has long been seen as a hallmark of the natural ageing process [1]. Quiescent stem cells have been identified in a wide range of adult tissues, capable of renewing and replenishing various organs, but the brain has long been exempt from this observation, with neural neogenesis believed to be exclusive to the developing embryo, ceasing shortly after birth [2–4] (for a comprehensive historical review [5]). Early indications of mitotic cells in rat brains were reported already at the turn of the last century [6, 7], and by the late 1960s Joseph Altman provided more evidence of cells undergoing active proliferation in the dentate gyrus, the olfactory bulb, and the neocortex of adult rats, cats, and guinea pigs [8–12]. Additional reports suggested neurogenesis in a range of animals, including songbirds [13–15], macaque [16], and humans [17, 18]. Major neurodifferentiation is now accepted to occur in specific niches of the subventricular zone (SVZ) of the lateral ventricles and the subgranular zone (SGZ) of the hippocampal dentate gyrus [19–23], and no or little post-natal neurogenesis in cortical neurons [24–26], although there is still a debate around adult neurogenesis [27–29]. However, the presence of quiescent neural stem cells (NSCs) questions their purpose, what regulates their activation and differentiation, and whether there is a connection between NSCs and the natural cognitive decline observed during human ageing. This review attempts to summarise our current understanding of what metabolic factors define and regulate neurodifferentiation in the adult brain.

The brain is a highly complex organ with mostly specialised cells, where neurons form a large interconnected network with synaptic activity, which is embedded in a complex set of glial cells. Astrocytes, a type of glial cells [30], are considered to play a supportive and protective role that provides the structural basis for our brain, as well as modulating synaptic transmissions [31, 32], provide energy for neurons [33], secretion and absorption of neurotransmitters, and can drive circadian behaviour [34]. Our ability to learn and adapt means also that this network is not static, but that interactions need to be maintained and new ones formed or replaced. This neuronal plasticity means cells will require different metabolic profiles during a variety of stages and understanding these requirements might allow us to improve their cellular function.

The developing brain follows a well-organised protocol during embryogenesis [35–38], and is considered to be predominantly metabolically glycolytic [39]. During adolescence the developing brain of both rats and humans has been shown to induce a metabolic shift from fatty acid oxidation to glucose-based metabolism [39–41], and by the time we are adults, our brains consume around 25% of our glucose intake, despite accounting for only ~ 2% of our body weight. At the same time, ~ 20% of our inhaled oxygen is used in the brain [39, 42–44], demonstrating a huge bioenergetic requirement, which is mostly satisfied by oxidative phosphorylation (OXPHOS) [45].

Nevertheless, the regions identified as containing NSCs have been shown to remain glycolytic [46], suggesting that although the purpose of embryonic and adult NSCs is very different, the mechanisms of differentiation might be similar. Structurally, the SVZ in the adult brain lines the lateral ventricles, separated by a layer of ependymal cells [46]. In rodents the neural precursors in the SVZ have been shown to form interneurones and astrocytes that will migrate to the olfactory bulb [47, 48]. Migration in humans is still debated, and relocation to the striatum or cortex have also been reported [49, 50]. The SGZ forms a narrow layer between the granule cell layer and hilus of the dentate gyrus in the hippocampus and is accepted to be one of the stem-cell-containing niches of the adult brain [51]. What exactly triggers neurogenesis is not fully established, but proliferation of NSCs in the SVZ and SGZ has, for instance, been observed as a consequence of ischemic stroke, leading to cell migration towards the lesion to contribute to repair [52, 53]. Thus, the SVZ and SGZ microenvironments are thought to provide the appropriate conditions for NSCs to proliferate, while also allowing for the differentiation into the relevant neurons via several rounds of asynchronous proliferation. This transformation has to undergo several distinct stages, where (a) the NSC has to exit its quiescent state, (b) proliferate, (c) migrate to its appropriate location, (d) terminally differentiate, and (e) integrate into the existing neuronal circuits [54, 55]. Each step is highly regulated and involves hormones, growth factors, neurotransmitters, and environmental factors. Additionally, diseases states or the genetic composition can influence greatly neurogenesis. Together these factors will inevitably affect the intracellular metabolic state, driving the different stages of neurogenesis.

## Energy metabolism in the brain

**Fig. 1:**
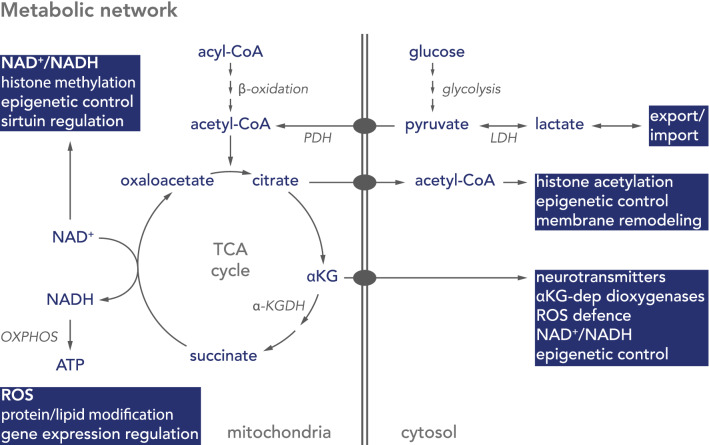
Schematic diagram of metabolic pathways important in neurogenesis. In astrocytes glucose is metabolised to pyruvate via glycolysis, metabolised by lactate dehydrogenase (LDH), and exported as lactate. Neurons take up astrocyte-derived lactate to convert it back to pyruvate. Pyruvate is imported into mitochondria and converted by the pyruvate dehydrogenase complex (PDH) to acetyl-CoA, which enters the citric acid (TCA) cycle. Acetyl-CoA is also generated by breakdown of fatty acids (acyl-CoA) during β-oxidation. The TCA cycle condenses oxaloacetate with acetyl-CoA to form citrate, which either acts as precursor for cytosolic acetyl-CoA or is metabolised in the TCA cycle to α-ketoglutarate (αKG). Mitochondrial NADH is oxidised by NADH-ubiquinone oxidoreductase (complex I) of the oxidative phosphorylation system (OXPHOS), while β-oxidation- or TCA cycle-derived FADH_2_ reduces ubiquinone via the electron transfer flavoprotein-ubiquinone oxidoreductase (ETF-QO) or succinate-ubiquinone oxidoreductase (complex II), respectively

Glucose metabolism begins with glucose entering the cell and being converted into pyruvate during glycolysis. During aerobic respiration, pyruvate enters mitochondria and is decarboxylated to acetyl-CoA by the pyruvate dehydrogenase complex (PDH), followed by condensation with oxaloacetate to citrate in the tricarboxylic acid (TCA) cycle (Fig. 1). In a series of reactions, citrate is then decarboxylated back to oxaloacetate, releasing carbon dioxide and reducing nicotinamide (NAD) and flavin (FAD) adenine dinucleotides to NADH and FADH_2_, respectively. The oxidation of these redox co-factors in the mitochondrial electron transport chain (ETC) leads to the formation of an electrochemical gradient across the inner mitochondrial membrane, which is used to drive an ATP synthase to form ATP from ADP and molecular phosphate (Pi) (Fig. 1) [56]. This oxidative phosphorylation (ETC + ATP synthase = OXPHOS) is dependent on oxygen, with approximately 80% of inhaled oxygen reduced to water at the final step of the ETC [42].

In the absence of oxygen pyruvate does not enter mitochondria but is converted to lactic acid by lactate dehydrogenase (LDH) (Fig. 1) [57]. This glycolytic metabolism of glucose is much less energy efficient, but some cells nevertheless rely predominantly on glycolysis to maintain their ATP pool, even in the presence of oxygen. This phenomenon is termed aerobic glycolysis and is important to provide precursors of essential biosynthesis pathways, such as purine and pyrimidines, amino acids, and triglycerides, which derive from intermediates of glycolysis and the pentose phosphate pathways [58–60]. In these cells the pyruvate kinase PKM2 regulates the last step within glycolysis, dephosphorylating phosphoenolpyruvate to pyruvate. By inactivating PKM2 intermediates of glycolysis accumulate and are preferentially channelled into other pathways [61]. This aerobic glycolysis is an important feature in cancer biology, where dividing cells rapidly need to increase their biomass [62]. Additionally, cataplerosis of the TCA cycle also diverts a number of intermediates to other pathways, reducing the contribution to energy production [63].

Besides glucose and lactate, ketone bodies represent an alternative fuel for neurons during fasting or extended exercise periods, reaching even 60–70% of the total energy supply for the brain [64]. Under low blood glucose levels adipocytes perform ketogenesis, releasing ketone bodies in the form of acetoacetate and *b*-hydroxybutyrate (BHB) into the bloodstream, which can cross the blood–brain barrier and be imported via MCT2 into neurons [65]. Additionally, unlike neurons, astrocytes are capable to perform ketogenesis providing with ketone bodies to neighbouring neurons [66]. Hence, neurons can shift from the oxidation of carbohydrates or lactate to ketone bodies in a transition process known as ‘G-to-K switch’ [67]. Evidence stems from mice either placed on intermittent fasting or high-ketone body containing diet, demonstrating the use of ketone bodies in the brain [67]. The effect of ketone bodies on neurogenesis is less clear, but has been correlated with improved synaptic plasticity, spatial learning, memory and cognition [68, 69]. Additionally, BHB has been shown to increase the expression of the brain-derived neurotrophic factor BDNF, and thereby promoting cellular resistance [70]. In contrast, prolonged hypothalamic exposure to ketone bodies stimulates hypothalamic neuropeptides and dysregulation of glucose homeostasis [71].

Astrocytes have a high glycolytic activity and are the main consumers of glucose in the brain, while only consuming around 20% of oxygen absorbed by the brain [39, 42–44]. In contrast, neurons are the main oxygen consumers, with high metabolic rate. This is achieved by instead of feeding the TCA cycle and utilising OXPHOS in mitochondria, astrocytes convert pyruvate to lactic acid via LDH and export it into the extra-cellular matrix via the monocarboxylate transporter, MCT4, where it is taken up by neurons via MCT2. There, lactate is converted back to pyruvate and used as fuel in mitochondria [72–75] or as signalling molecule [33, 76]. Astrocytes thus act as a buffer, safeguarding neurons from fluctuating blood glucose levels by continuously fuelling lactate to neurons. And indeed, neurons have been shown to have low glycolytic activity [77] and preferentially use lactate [75, 78], although both glucose and lactate have been shown to be able to stimulate oxidative metabolism in mature neurons [75, 79]. The energetic buffer capacity of astrocytes is further supported by the observation that unlike neurons, astrocytes have the capacity to store glycogen, which is broken down back to glucose by glycogenolysis upon synaptic activity [74, 80].

## Metabolism in neural stem and progenitor cells

Hence, stem cells are generally considered to be glycolytic [81–83], and in agreement NSCs in the SGZ and SVZ are considered to have predominately glycolytic activity. This glycolytic nature of stem cells had for long been attributed to a combination of their cellular demands and a hypoxic microenvironment, but it has become clear that stem cells are indeed capable of using OXPHOS, but require glycolysis to maintain stemness rather than being an adaptation to its environment [84–88]. On the other hand, the oxidation of fatty acids has also been suggested to function as fuel source in NSCs, especially during NSC activation [89–94], and this activation of NSCs is dependent on a functional ETC and OXPHOS [95, 96]. Furthermore, the ability to adapt their metabolic state seems to be essential in NSCs for proliferation and differentiation [97–99]. Growing evidence suggests that mitochondria play a central and driving role in this transition, not only by providing ATP but also by regulating individual steps during neuronal differentiation, such as managing the cellular redox state, intracellular signalling pathways, or the epigenetic state of the cell [100–103]. Accordingly, to accommodate the changing metabolic demands of activation and differentiation to a neuronal lineage, NSCs need to switch their metabolism from a predominantly glycolytic to one that utilises mitochondrial OXPHOS.

## The metabolic switch in neurogenesis

**Fig. 2:**
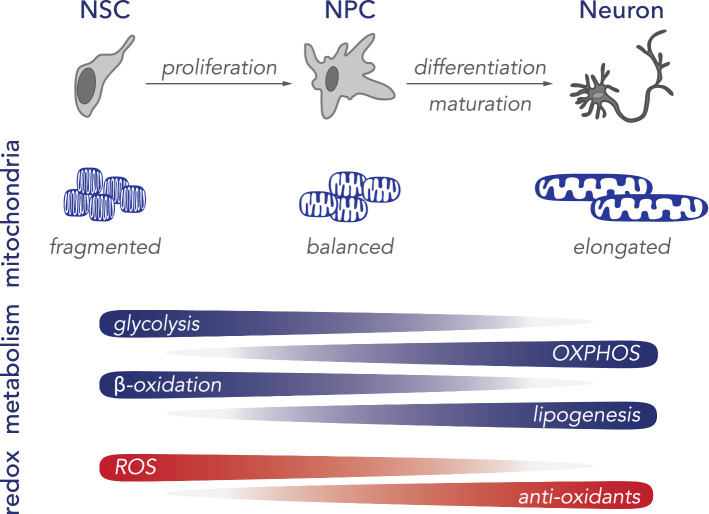
Schematic diagram of neuronal differentiation. During neurogenesis, neural stem cells (NSC) need to proliferate to neural progenitor cells (NPC) and differentiate into mature neurons. This progression is accompanied by several shifts, including the activation and proliferation of mitochondria, a transition from a glycolytic to aerobic metabolism, relying on oxidative phosphorylation (OXPHOS) for ATP synthesis. Additionally, fatty acid metabolism shifts from energy metabolism to de novo lipogenesis. The increased metabolic activity of the cells is accompanied by reactive oxygen species (ROS), which is countered by the activation of oxidative stress response genes to reduce overall ROS levels

A number of reports have demonstrated that the transition from NSC to a neuronal lineage is accompanied by increased mitochondrial biogenesis, as well as the downregulation of glycolysis and fatty acid oxidation pathways (Fig. 2) [90, 91, 93, 95, 97, 99, 104, 105]. For instance, the progression from pluripotent progenitor cell to neuron is characterised by a strong reduction in glycolysis-related proteins, such as hexokinase 2 (HK2) and isoform A of lactate dehydrogenase (LDHA), which metabolises the reduction of pyruvate to lactate. Additionally, a switch from PKM2 to its constitutively active isoform PKM1 and an upregulation of OXPHOS-related genes has been observed [97, 99].

Several factors have been shown to be important during this transition. For instance, the TP53-inducible glycolysis and apoptosis regulator TIGAR was shown to increase during brain development and neuronal development [106]. TIGAR inhibits glycolysis, directing the pathway into the pentose phosphate shunt, and is also proposed to regulate HK2 activity. Silencing in NSCs resulted in reduced neuronal differentiation and decreased expression of neuronal markers, such as β-III tubulin, microtubule-associated protein 2 (MAP2), glial fibrillary acidic protein (GFAP), Ngn1, and NeuroD1. Moreover, TIGAR expression was accompanied by decreased lactate production and increased expression of the neuron-specific lactate dehydrogenase B (LDHB), thus, shifting towards pyruvate production and mitochondrial metabolism [106]. Downregulation of HK2 and LDHA is required for neurodifferentiation, and overexpression of either HK2 or LDHA in NPCs blocks neurodifferentiation and promotes astrocyte formation, thus guiding differentiation away from a neuronal towards a glial profile [97].

Directionality of LDH seems to be predominantly regulated by substrate/product concentrations, although LDHA is proposed to prefer the conversion of pyruvate to lactate, while LDHB catalyses the reverse reaction [107]. LDHA is regulated by the transcription factors c-MYC and the hypoxia inducible factor HIF1α, but a recent study, using a neuroepithelial stem cell (NES) model of the autophagy adaptor Sequestosome 1 (SQSTM1/p62), suggested that upregulation of LDHA was independent of both c-Myc and HIF1α, arguing for additional pathways [99]. P62 is a cytosolic, multi-domain protein, considered to be involved in, among others, selective autophagy [108]. Patients with bi-allelic null mutations in p62 present with childhood- or adolescence-onset neurodegenerative disorder, characterised by progressive gait abnormalities, ataxia, dysarthria, dystonia, vertical gaze palsy, and cognitive decline [109, 110]. Loss of p62 in NES cells resulted in a dramatic increase of LDHA expression, which correlated with deficient neurodifferentiation [99]. This increase was absent in patient-derived fibroblasts, suggesting that the upregulation of LDHA might be stem cell-specific [99]. NES cells are a proliferative neural stem cell line that displays a high differentiation potential to various neuronal subtypes and glial cells [111]. Although patient-derived NES cells arrested proliferation upon neuronal induction, they failed to fully differentiate into neurons, as indicated by lower levels of the neuronal markers β-III tubulin and HuC/D. However, this did not seem to be due to the absence of mitochondrial-specific autophagy, but rather due to the cells’ failure to fully commit to a more aerobic metabolic profile as indicated by an inability to upregulate genes important for increased mitochondrial function, such as OXPHOS-specific genes. This could be partially rescued using the antioxidant N-acetylcysteine (NAC), suggesting an important role for p62 in oxygen sensing or reactive oxygen species (ROS) management [99]. Roles for p62 in oxidative stress management [112], hypoxia [113], and more recently, redox state management [114], have previously been suggested, with p62 shown to regulate the nuclear transcription factor erythroid 2-related factor 2 (NRF2). Under oxidative stress conditions p62 stabilises NRF2 by preventing its KEAP1-mediated degradation, which then translocates to the nucleus to bind to and activate upstream promoter regions of genes involved in inflammatory or antioxidant responses [115–119]. Two oxygen-sensitive cysteine residues in p62 have recently been suggested to activate autophagy in response to oxidative stress [114]. P62 is therefore potentially central to coordinating redox state and protein homeostasis in neurogenesis [99].

Support comes from rodent models deficient of p62, which present with several neurological phenotypes, ranging from memory loss to behavioural abnormalities to the accumulation of Tau tangles [120–123]. Specifically, p62 was shown to be important for neuronal cell survival and development in rats [121], while the deletion of p62 rescued the NSC pool in the SVZ and dental gyrus of autophagy-deficient FIP 200-KO mice, demonstrating an important role for p62 in the regulation of neuronal development, probably by regulating intracellular superoxide levels [124].

## Redox state and ROS

**Fig. 3:**
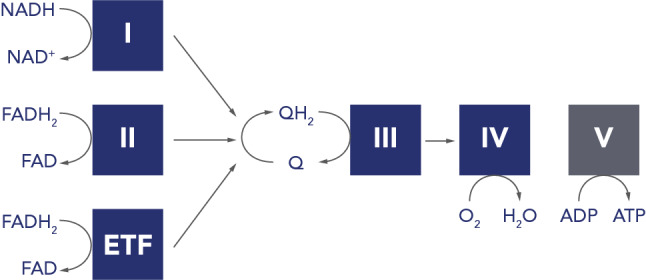
Schematic diagram of the reduction of ubiquinone. The complete oxidation of pyruvate generates 4 NADH molecules and 1 FADH_2_, which are oxidised on complex I (I; NADH: ubiquinone oxidoreductase) and complex II (II; succinate: ubiquinone oxidoreductase) of the mitochondrial electron transport chain, respectively, while reducing ubiquinone (Q) to ubiquinol (QH_2_). In contrast, one round of β-oxidation also forms 4 NADH, but 2 FADH_2_. The second FADH_2_ is oxidised by the electron transfer flavoprotein: ubiquinone oxidoreductase (ETF), which also contributes to the QH_2_ pool. Additionally, the two mitochondrial dehydrogenases glycerol 3-phosphate dehydrogenase and dihydroorotate dehydrogenase (both not shown) can contribute to the QH_2_ pool as part of glycerol metabolism and the de novo pyrimidine biosynthesis pathway, respectively. QH_2_ is oxidised at complex III (coenzyme Q: cytochrome c oxidoreductase). Oxygen is reduced to water at complex IV (IV; cytochrome c oxidase). An ATPase synthase (V) synthesises ATP from ADP at Pi)

Oxygen levels are crucial in determining cell fate [125], not only by regulating specific gene expression profiles through transcription factors such as HIF1α, but also by directly influencing enzymatic reactions [126]. In this respect, mitochondria are the largest consumers of oxygen, reducing it to water in the ETC in a coordinated fashion. Reactive oxygen species (ROS) are reduced forms of molecular oxygen that are predominantly formed during the transition of electrons in the ETC in mitochondria, or in peroxisomes. During OXPHOS a small percentage of molecular oxygen is reduced to superoxide (·O^−^_2_) due to electron leakage at complexes I or III of the mitochondrial respiratory chain (Fig. 3) [127]. Further dismutation or reduction can lead to a number of different radical oxygen species, including hydrogen peroxide (H_2_O_2_), which can leak out of mitochondria and act as a signalling molecule [128, 129]. Thus theoretically, ROS levels act as a function of mitochondrial respiration. However, multiple factors influence ROS levels, including oxygen availability, redox states of the redox co-factors NADH, FADH_2_, or ubiquinone, activities of antioxidant factors, such as glutathiones or superoxide dismutases, mitochondrial morphology, as well as mutations in OXPHOS subunits [130], and ROS levels are therefore much more complicated. Nevertheless, ROS can spontaneously react with a range of biological materials, including lipids, proteins, or nucleic acids, altering their function, and under stress situations ROS levels can lead to severe cellular damage, induce apoptosis and cell death, and has been suggested to be a part of various pathologies and the natural ageing process [131, 132].

ROS levels are known to be able to influence and activate various gene expression profiles [133] and have been suggested to be important for stem cell fate [134]. For instance, culturing NPCs in the presence of antioxidants improves neurodifferentiation [135, 136], and regulation of ROS levels has been suggested to be important for proliferation of hippocampal NPCs in mouse embryos [137]. Additionally, an age-dependent reduction in NRF2 expression has been linked to a reduced capacity of NPCs to regenerate in the SVZ of rats [138], but not in the SGZ of the dentate gyrus [139]. Nevertheless, NRF2 activity was important in both niches for linage commitment of NPCs to either a neuronal or glial fate. Further, studies in mice suggest that NRF2 levels can affect mitochondrial membrane potential and OXPHOS function, with loss of NRF2 associated with increased glycolytic activity and reduced substrate availability for mitochondrial respiration [140]. Thus, reduced NRF2 levels correlate with the age-associated reduction in NSCs in murine brains [138], and recent data suggest that this is caused by an inflammatory response in the ageing brain, linked to increased quiescence to protect the NSC pool [141]. The authors were also able to reactivate these old NSCs, suggesting a potential treatment target for age-associated neurodegeneration [141].

In general, quiescent NSCs are thought to have higher ROS levels, which gradually decrease during the progenitor stage, with low levels of ROS reported in mature neurons [128]. A recent study in mice suggested that NSCs require a spike in ROS levels before committing to proliferation and that this induction is prior to the redirection of cellular lipid metabolism to lipogenesis or induction of mitochondrial biogenesis [142]. Interestingly, the authors also suggested that NSCs can shift between different levels of proliferation induction, depending on ROS levels, without fully committing to enter neurogenesis [142]. It is therefore likely that multiple signals have to come together to initiate neurogenesis.

Oxygen levels vary across the brain, allowing for differential responses across neuronal niches. HIF1α is a main sensor of cellular oxygen, and upon low oxygen concentrations is stabilised, leading to the activation of a hypoxia response gene expression program [143–146]. Under hypoxic conditions the hydroxylation of HIF by α-ketoglutarate-dependent dioxygenases is suppressed, leading to the stabilisation of HIF1α, translocation to the nucleus and activation of gene expression. α-ketoglutarate (αKG, also known as 2-oxoglutarate) is a TCA cycle intermediate that can be converted to the neurotransmitter glutamate [146], thus firmly linking energy metabolism and oxygen sensing to neuronal function and potentially neurogenesis [147]. Recently, chronic mild hypoxia has been linked to adult neurogenesis in the hippocampus [148], while the deletion of HIF1α in mouse neural cells led to hydrocephaly, reduced number of NSCs and impaired spatial memory [149]. Along this line, work on stroke patients suggests that ischemia leads to the proliferation of NSCs in the SVZ and SGZ of the dentate gyrus, migration towards the lesion, and integration into the damaged area [52, 53]. Thus, oxygen sensing is clearly an important feature during neurogenesis, and its regulation is not only required for NSCs to exit their quiescent state but also to commit to their final cell linage. In agreement, oxygen regulates stemness via Wnt/β-catenin signalling [150], although this has been suggested to be independent HIF1α [151].

## Lipid metabolism and neurogenesis

Long chain saturated fatty acids contain almost double the energy compared to glucose, and the brain consists of the second highest lipid content in the body after adipocytes. Despite this, fatty acid oxidation is low in the brain [152]. This is in contrast to other tissues with high energy demand, such as the heart, which utilise fatty acids as energy source, and the brain presumably requires fatty acids for lipid biosynthesis, rather than as energy source. While glucose is readily absorbed by cells from the blood, fatty acid transport is coupled to albumin, which does not cross the blood brain barrier, limiting lipid availability in the brain [153]. Nevertheless, the brain requires large amounts of lipids for membrane formation, which cannot be explained by de novo lipogenesis alone, and specific transporters for the uptake of essential fatty acids into the brain have since been identified [154–157]. There are several indications that astrocytes use β-oxidation as fuel source, allowing glucose to be predominantly used to support neurons [153, 158], but as β-oxidation releases acetyl-CoA there might be a metabolic compartmentalisation in neurons that does not favour lipids as energy source. For instance, acetyl-CoA, can either enter the TCA cycle, or be used for histone acetylation to regulate gene expression. It can form ketone bodies, which are used as energy source in neurons during starvation or enter the mevalonate pathway to generate farnesyl-pyrophosphate, which is important for the biosynthesis of the redox co-factor ubiquinone, sterol, cholesterol, heme A, dolichols, or the prenylation of proteins (Fig. 1) (For review see [158]). There are several other factors arguing against the use of fatty acids as energy source in neurons. First of all, the brain often requires fast bursts of ATP, which cannot be achieved by β-oxidation; further, β-oxidation has a higher oxygen requirement, which constitutes an increased risk for neurons to become hypoxic; and thirdly, the breakdown of fatty acids increases the FADH_2_/NADH ratio, due to FAD reduction at the electron transfer flavoprotein-ubiquinone oxidoreductase (ETF-QO), leading to increased competition for the ubiquinone pool in the inner mitochondrial membrane (Fig. 3). This, in turn, can depolarise mitochondria and increase the risk for ROS production at complex I of the ETC.

Nevertheless, as mentioned above, the glycolytic activity of NSCs is used to drive the synthesis of biomaterials for cell sustainability, with high expression of regulatable PKM2 [61], and NSCs in the SVZ of adult mouse brains have been shown to use β-oxidation for energy production [91, 92]. The increased bioenergetic demand during neuronal differentiation is accompanied by a switch to a more glucose-based metabolism, with a down-regulation of fatty acid oxidation. Simultaneously, de novo lipogenesis is upregulated [94, 153]. NSCs express a number of different fatty acid binding proteins (FBPs), with FBP5 and 7 important for NPC differentiation and migration [153, 159–161]. This correlates with an increase in lipid synthesis through fatty acid synthase during maturation to allow for increased membrane lipid synthesis [104]. This shift is regulated by the thyroid hormone responsive protein SPOT14, which is highly expressed in NSCs [90] and inhibits the fatty acid synthase FASN [104]. Additionally, metabolic analysis of embryonic NPCs demonstrated an increase in long chain fatty acids, citrate, cholesterol synthesis and decreased acyl-carnitines, suggesting fatty acid synthesis and membrane remodelling [105]. Thus, de novo lipogenesis is an important requirement for NSCs to exit their quiescent state and initiate proliferation. Interestingly, lipid droplets secreted from glial niche cells in Drosophila melanogaster have been shown to protect NSCs from exogenous ROS and enter proliferation [162], suggesting a further function of glial cells in regulating neurogenesis.

## Mitochondrial morphology during neurodifferentiation

The increase in mitochondrial abundance and function is accompanied by an increase in mitochondrial DNA levels [163, 164], mitochondrial gene expression [165], and activity [166]. A reduced neuronal differentiation could be observed in culture by inhibiting mitochondrial translation with chloramphenicol [105, 165]. In addition, the expression of two master regulators of mitochondrial biogenesis, the peroxisome proliferator-activated receptor gamma co-activator 1-alpha (PGC1α) and the oestrogen-related receptor gamma (ERRγ), precedes increased mitochondrial abundance [97, 164]. This activation of mitochondria is marked by a remodelling and replacing the mitochondrial network [167].

Exercise is a well-known activator of mitochondrial biogenesis and has been suggested to trigger adult neurogenesis [168, 169]. However, overexpression of PGC1α in skeletal muscle had no effect on age-associated decline of NSCs [170], despite improved muscle performance and increased levels of the neuroplasticity promoting brain-derived neurotrophic factor, BDNF [168]. Thus, the exact mechanism is not yet clear. Nevertheless, the activation of stem cells coincides with changes in mitochondrial morphology from rounded small mitochondria, with dense and compact cristae in quiescent NSCs, to a more open and structured network [167]. These changes are believed to be fundamental to neurogenesis [91, 133, 171–173], and are possibly regulated by ROS signalling [133]. The importance of mitochondrial fusion and fission is well established, with numerous models, as well as mutations in patients with mitochondrial disease, demonstrating that disrupting either process can lead to severe consequences in the brain [174]. Additionally, the selective removal of mitochondria via autophagy, also termed mitophagy, has been shown to be an important contributor to cellular metabolism [175–177]. For instance, reduced expression of pro-apoptotic factors in murine NSCs resulted in reduced neurogenesis [178–180], while levels of the neuronal development regulator, NOTCH, have been suggested to be regulated by autophagy [181].

Many of the factors described above are essential for cell survival and the described mechanisms often require models with cell-type specific disruption of the factor in question. Although these studies have provided important information regarding the mechanisms of neurogenesis, it is likely that many essential genes will have similar effects, and thus the importance of, for instance, mitochondrial function in driving neurogenesis remains to be established. An indication that this is indeed the case stems from a mouse model with deficient proof-reading ability of the mitochondrial DNA polymerase γ, POLG [182, 183]. These mice present with increased mtDNA mutations, an age-associated decline in mitochondrial function, and a progressive aging phenotype [182, 184]. Ahlqvist and colleagues demonstrated that increased mtDNA mutation loads correlated with a reduction in NSCs in the SZV of adult mice, reduced self-renewal capacity, and decreased mitochondrial respiration [185]. Interestingly, self-renewal could be partially restored by NAC treatment, suggesting that ROS or the cellular redox state is important for NSC self-renewal [185]. Indeed, an increase in mtDNA mutations, together with reduced mitochondrial function, has been observed in brains during the natural ageing process in humans [186], and the prospect that modulating mitochondrial function and/or redox state to improve NSC renewal is enticing. These findings are supported by the observation that supplementation of somatic stem cell cultures with NAD^+^ improves cell survival and mitochondrial function [187, 188].

The requirement of NSCs to control their ROS and redox states suggests that oxygen might have negative effects in conjunction with mitochondrial disease [126]. In this regard, two mouse models with mitochondrial disease, could be rescued by reducing breathing oxygen levels [189, 190], although just increasing HIF1α had no effect [191]. Neural cells were not investigated in these models, but it will be interesting to determine whether hypoxia treatment has a positive effect on neurogenesis in combination with a mild mitochondrial dysfunction [192].

## Conclusions

Several pathologies have been correlated with impaired neuronal differentiation, and the age-associated decline in cognitive function has been attributed to reduced neurogenesis with age [184]. For example, the autism spectrum disorder (ASD) has been linked to impaired neuronal development, since patient-derived reprogrammed cells showed impaired neuronal maturation [193], while a reduction of neurogenesis and impaired differentiation into neurons was reported in epileptic patients [194, 195]. Interestingly, reduced adult hippocampal neurogenesis has recently been observed in patients with Alzheimer’s disease [23], and mitochondrial dysfunction is a key feature in Alzheimer’s pathology [196], further pointing to an important connection between neurogenesis and mitochondria.

Over a century ago Santiago Ramon y Cajal wrote that in the brain "everything may die, nothing may be regenerated. It is for the science of the future to change, if possible, this harsh decree" [4]. The identification of adult neurogenesis has provided us with the possibility, but we are only at the beginning of understanding what factors are involved in the proliferation and differentiation of neuronal stem cells. Metabolism clearly plays an important part in this development and it is also clear that an elaborate network of factors, ranging from metabolites, oxygen, and transcription factors is required to carefully regulate quiescence, proliferation, and differentiation. The prospect that combinations of redox state modifiers, oxygen levels, and diet [197] has already shown to have positive effects on activating NSCs, brings hope to that the future indeed will be able to change this "harsh decree".
